# Pre-hospital and admission parameters predict in-hospital mortality among patients 60 years and older following severe trauma

**DOI:** 10.1186/1757-7241-21-91

**Published:** 2013-12-21

**Authors:** Miklosh Bala, Dafna Willner, Dima Klauzni, Tali Bdolah-Abram, Avraham I Rivkind, Mahmoud Abu Gazala, Ram Elazary, Gidon Almogy

**Affiliations:** 1 Department of Surgery and Trauma Unit, Hadassah University Hospital, Ein Kerem, pob 12000, Jerusalem, 91120, Israel; 2 Department of Anesthesiology and Intensive Care, Hadassah University Hospital, Hadassah Hospital, pob 12000, Jerusalem, 91120, Israel; 3 Department of Social Medicine, Hebrew University, Hadassah Hospital, pob 12000, Jerusalem, 91120, Israel

## Background

The world population of inhabitants of greater than 60 years of age has doubled since 1980 and is predicted to reach 2 billion by 2050 [1]. Trauma is the sixth leading cause of death in patients over 60 years of age [2]. Though this growing elderly population only comprises 12% of overall trauma patients, they consume considerable medical resources [3] and are more likely to require hospital admission [4]. An aggressive approach should be established throughout the management of the elderly trauma patient in order to reduce mortality and the incidence of permanent disability [5]. In the past decade, the overall mortality rate due to trauma decreased. However, in the elderly population (>65 years of age), the incidence of trauma-related mortality is still high, mostly secondary to falls [6]. Elderly patients have a higher mortality after trauma as well as a higher complication rate, specifically for pulmonary and infectious complications [7,8].

Trauma in the elderly clearly poses special challenges to the physician, with physiological changes of age impacting morbidity and mortality. Notwithstanding, little information is available regarding risk factors that aid in predicting increased mortality in this population. More so, there are significant findings in the literature showing that severely injured geriatric trauma patients who do survive their hospitalization have appreciable long-term survival and return to independent living [9-11].

Our study is a retrospective review of our experience with severely injured elderly patients. Our primary objectives were to describe the different pattern of injury among the elderly and define and analyze predictors of in-hospital mortality. Our secondary objective was to determine whether pre-existing co-morbidities had an adverse effect on outcome.

## Methods

Hadassah University Hospital, Ein Kerem Campus, is a tertiary medical center and the only level I trauma center in the Jerusalem vicinity. Emergency medical services (EMS) in Israel are provided by a government funded national organization with regional control. The catchment area incorporates Jerusalem and nearby towns and villages and includes a population of approximately one million inhabitants.

Inclusion criteria to the study included all trauma patients ≥ 60 years of age who presented to our Level I Trauma Center with an injury severity score (ISS) ≥16 between January 2006 and December 2010. Patients who were pronounced dead at the trauma bay or had a do not resuscitate order were excluded from the study. Data was retrieved from medical records and the trauma registry database. The trauma registry is a prospectively collected database that is updated daily by dedicated personnel and has institutional review board (IRB) approval.

All charts were retrospectively reviewed for demographics, ISS, GCS (Glasgow Coma Scale) at presentation to the emergency department (ED), mechanism of injury (MOI), body regions injured, pre-existing co-morbidities, intensive care unit length of stay (ICU LOS), hospital LOS, surgical interventions, complications, and in-hospital mortality. Patients were divided into 3 age groups: 60-69 years, 70-79 and ≥80. The main outcome measure was in-hospital mortality. This was defined as death which occurred at the trauma center. In order to avoid missing late deaths which were directly related to the trauma, we chose to include patients who were discharged from the hospital but died within 30 days of the traumatic insult regardless of patient location at time of death. Co-morbidities were defined as presented in Table 1.

**Table 1:** Definition of co-morbidities

	
Cardiac disease	Known history of ischemic heart disease, previous cardiac interventions for ischemic heart disease
Malignancy	Currently under oncological follow up for active oncological disease
Diabetes mellitus	Patient requiring insulin or oral hypoglycemic therapy
Neurological disease	History of cerebrovascular accident, severe parkinsonism or antiepileptic therapy
Dementia	A patient with an established diagnosis of dementia
Hypertension	History of hypertension requiring medication
Chronic anticoagulation	Patients currently on anticoagulation (low molecular weight heparin or Warfarin), and anti-platelet therapy (excluding aspirin)
Chronic renal failure	Preexisting renal insufficiency on admission
Chronic pulmonary disease	Ongoing treatment for chronic obstructive pulmonary disease or asthma

### Statistical analysis

Data are presented as mean ± standard deviation. The Fisher's exact test was used to compare proportions and the Kruskal-Wallis test was used to compare continuous non-parametric variables between the three groups. The chi-squared test for trends was used to compare mortality between the different age groups. A logistic regression model was used to define predictors of death. In order to determine predictors of in-hospital death, parameters which were significant on univariate analysis were entered into a stepwise, forward regression model. A p value of 0.05 or less was considered statistically significant. Statistical analysis was performed using IBM SPSS Statistics (IBM Corp. Released 2011. IBM SPSS Statistics for Windows, Version 20.0. Armonk, NY: IBM Corp.)

## Results

### Patient population

There were 417 trauma patients older than 60 years of age and with an ISS ≥16 who presented to our trauma unit during the study period and who met the inclusion criteria. One patient was excluded due to incomplete data. The mean age of the study cohort was 76.9 years (±9.6) and there were 232 males (55.6%). 174 patients (41.7%) were ≥ 80 years. Mean ISS for the whole cohort was 22.9 (±8.4). 230 patients (55.3%) had an ISS of 16 to 24 and 186 patients (44.7%) had an ISS ≥25.

The demographic and clinical characteristics of the patients according to the age groups are noted in Table 2. The rate of males declined significantly with age. Although ISS and the median number of regions injured decreased significantly with age (both p < 0.001), mortality increased significantly. The most common MOI for all patients was falls (n = 271, 65.1%) followed by pedestrian injuries (n = 71, 17.1%), motor vehicle collision (MVC) (n = 49, 11.8%), burns (n = 11, 2.6%), and other causes (assault, penetrating trauma, etc, n = 15, 3.6%). The MOI was different between the age groups. The rate of fall as a MOI increased significantly with age (p < 0.0001) while the rate of patients involved in MVC either as pedestrians or in a vehicle decreased significantly with age (p < 0.0001).

**Table 2:** Univariate analysis of demographic and clinical data by age groups

	**Age 60-69 (n = 105)**	**Age 70-79 (n = 137)**	**Age ≥ 80 (n = 174)**	**P value †**
Age, years	64.2 ± 3.0	74.9 ± 2.9	86.1 ± 4.9	
Gender, males *	71 (67.6)	82 (59.9)	79 (45.4)	<0.0001
Mechanism of Injury (fall) *	42 (40.0)	86 (62.8)	143 (82.2)	<0.0001
ISS ≥ 25 *	56 (53.3)	58 (42.3)	70 (40.2)	0.04
GCS upon arrival ≤8 *	24 (22.9)	23 (16.9)	30 (17.2)	NS
Head AIS ≥ 3 *	58 (55.2)	97 (70.8)	152 (87.4)	<0.0001
Number of regions injured	2.61 ± 1.5	2.09 ± 1.4	1.55 ± 0.9	<0.0001
Intubation upon arrival *	16 (15.2)	16 (11.7)	20 (11.5)	NS
Required surgery *	52 (49.5)	52 (38.0)	52 (29.9)	0.0008
ICU admission *	71 (67.6)	74 (54.0)	81 (46.6)	0.0009
Complications *	30 (28.6)	24 (17.5)	35 (20.1)	NS
Mortality *	10 (9.5)	23 (16.8)	41 (23.6)	0.003

Data are presented as mean (and SD) or number of patients (and percentage points) *.

† Chi-squared test for trends.

*ISS* Injury severity score, *GCS* Glasgow coma scale, *AIS* Abbreviated injury scale, *ICU* Intensive care unit.

### Types of injuries

Head and facial trauma (median AIS = 4, range 1-5) was the most common type of injury (339/416 patients, 81.5%). Thoracic injuries occurred in 39.7% (165/416) of the patients, with rib fractures and/or flail chest being the most common thoracic injuries (88/165, 53.3%). Cervical spine injuries were present in 9.6% (40/416) of patients. Thoracic, lumbar, and sacral spine injuries were present in 10.1% (42 of 416) of patients. Abdominal injuries were present in 8.9% (37/416) of patients. Pelvic fractures were present in 12.5% (52/ 416) of the patients and long bones fractures in 20.7% (86/416).

**Figure 1:**
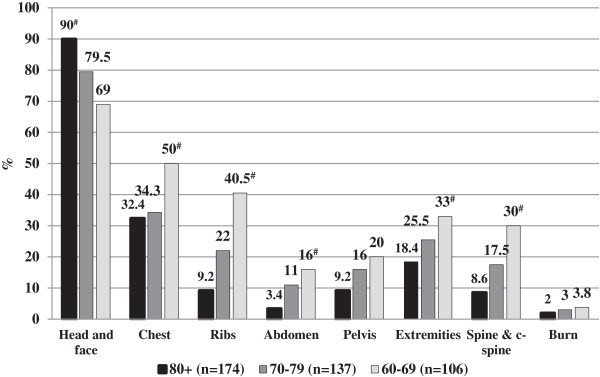
**Injury patterns according to the different age groups (data shown as percentage points). **^#^ p < 0.05 Chi squared test for trends.

Figure 1 shows the injury patterns according to age groups. Head and facial injuries were significantly more common in the ≥80 age group compared with the other groups (p = 0.0006). Injuries to the torso, extremities and spine were significantly less common in the ≥80 age group.

### Co-morbidities

The impact of pre-existing co-morbidities on outcome was compared between patients who died and those who survived. On univariate analysis, only chronic renal failure (CRF) was associated with a poorer outcome (11 patients [14.9%] in the mortality group vs. 20 patients [4.8%] in the survival group, p = 0.013). Analysis of co-morbidities by the different age groups (60-69, 70-79 and ≥80) showed that hypertension (HTN) (16.2%, 43.1% and 49.4%, respectively, p = 0.035) and the chronic use of anti-coagulant treatment (4.8%, 9.5% and 12.6%, respectively, p = 0.018) were more common in the older age groups. Not surprisingly, the mean number of co-morbidities per patient increased significantly with age (0.77, 1.3, and 1.4, respectively, p = 0.023).

### Complications

Eighty nine patients (21.4%) developed a total of 143 complications during the course of their hospital stay. Pulmonary complications were the most common and included pleural effusions (32, 7.7%), pneumonia (21, 5.0%) and atelectasis (20, 4.8%). Non-pulmonary complications included renal failure (11, 2.6%), septicemia (5, 1.2%) and wound infection (4, 1.0%). The total number of complications in the mortality group was significantly higher compared to the survival group (33 complications for 74 patients [44.6%] vs. 110 complications for 342 patients [32.2%], respectively, p = 0.044]. There were 62 patients (18.1%) who developed pulmonary complications and 24 patients (7.0%) who developed infectious complications in the survival group compared with 11 patients (14.9%) with pulmonary and 7 patients (9.5%) with infectious complications in the mortality group (differences not significant). The number of patients who developed complications was similar between the different age groups (30 patients [28.6%] with complications in the 60-69 group, 24 patients [17.5%] in the 70-79 group and 35 patients [20.1%] in the ≥80 group, p = 0.146) (Table 2).

### In-hospital mortality

In-hospital mortality rate was 17.8% (74/416). One patient who died (1.4%) was discharged from the hospital but died within 30 days of the trauma and was therefore defined as hospital mortality. Thirty-three of the patients (5.5%) died within the first 24 hours of admission to our unit. Univariate analysis of clinical parameters is shown in Table 3. An increased mortality rate was observed in the ≥80 group compared with their younger counterparts and this was statistically significant (23.6% [41/174], vs. 16.8% in the 70-79 group [23/137], and 9.5% in 60-69 group [10/105], p = 0.003) (Table 2). MOI did not appear to affect mortality rate, with the exception of burns which had the highest mortality (7/11 patients, 64.6%). All patients with burns over ≥20% of body surface area died.

**Table 3:** Univariate analysis of demographic and clinical parameters according to in-hospital outcome

	**Mortality group (n = 74)**	**Survival group (n = 342)**	**P value**
Age, years	79.8 ± 8.9	76.2 ± 9.7	0.003
Gender, males *	45 (60.8)	187 (54.7)	NS
ISS ≥ 25*	62 (83.8)	124 (36.3)	<0.0001
Mechanism of injury-fall *	47 (63.5)	224 (65.5)	NS
Number of regions injured	2.0 ± 1.5	1.99 ± 1.3	NS
Intubation upon arrival*	26 (35.1)	26 (7.6)	<0.0001
GCS upon arrival ≤8 *	38 (51.4)	40 (11.7)	<0.0001
Head AIS ≥ 3 *	58 (78.4)	250 (73.1)	NS
Creatinine at arrival (mg %)	136.1 ± 121.6	96.2 ± 52.3	<0.0001
INR at arrival	1.54 ± 0.9	1.18 ± 0.4	<0.0001
Required surgery *	30 (40.5)	127 (37.1)	NS
Required ICU stay *	53 (71.6)	173 (50.6)	0.001
Received blood products *	50 (67.6)	159 (46.5)	0.001
Complications *	19 (25.7)	70 (20.5)	NS

Data shown as mean (and SD), or number of patients (and percentage points)*.

*ISS* Injury severity score, *GCS* Glasgow coma scale, *AIS* Abbreviated injury scale, *INR* International normalized ratio, *ICU* Intensive care unit.

### Predictors of in-hospital mortality

Multivariate analysis was performed to analyze predictors of in-hospital death. Variables which were significant on univariate analysis (Table 3) were entered into a stepwise forward regression model. Variables entered included age, ISS, CRF, number of co-morbidities, intubation upon arrival at the trauma unit, GCS upon arrival, international normalized ratio (INR) at arrival, and the requirement for ICU and/or blood transfusion. Older age, CRF, low GCS, high INR, and the need for intubation were all found to be predictors of in-hospital mortality (Table 4). In order to underline the clinical relevance of our results, we applied well-established clinical cutoff points and categorized age (<80 and ≥80), INR (<1.2 and ≥1.2), and GCS (3-8, 9-13, and 14-15). Subsequently, age ≥ 80 (OR 2.29), GCS < 14 upon admission (OR 4.12), intubation in ED (OR 3.32), CRF (OR 3.65) and INR ≥ 1.2 (OR 3.53) were found to be significant predictors of in-hospital mortality.

**Table 4:** Multivariate analysis of predictors of in-hospital mortality

	**Adjusted OR**	**95% CI**	**p value**
Age, years	1.08	1.04-1.12	<0.0001
GCS upon admission	0.81	0.75-0.87	<0.0001
Intubation in ED	4.33	1.77-10.57	0.001
CRF	3.49	1.35-8.98	0.01
INR	2.38	1.45-3.91	0.001

*OR* Odds ratio, *CI* Confidence interval, *GCS* Glasgow coma scale, *ED* Emergency department, *CRF* Chronic renal failure, *INR* International normalized ratio.

## Discussion

A six times greater mortality rate has been reported in elderly patients compared to younger trauma patients when taking into account the degree of injury [12]. We analyzed data on severely injured geriatric trauma patients treated at our level I trauma center. The major finding of this study is that pre-hospital parameters of age, and CRF, and initial in-hospital findings of a low GCS, the need for intubation, and an elevated INR can predict in-hospital mortality following severe trauma in an elderly population.

There is no consensus in the literature regarding the definition of a geriatric trauma patient and the age varies from 55 to 70 years old [13]. Some have defined geriatric trauma as patients over the age of 65 years corresponding to retirement policies. Furthermore, the rapidly increasing demographic changes in the elderly population over 80 years of age have led to a differentiation between old and the very old [14]. As previously been done, we divided our patients into 3 age categories: 60-69, 70-79 and ≥80. Our results show that the pattern of injury shifts gradually with these age groups.

The overall severity of the trauma load and its impact on the patient’s anatomy and physiology is usually expressed as an ISS of ≥ 16 [15]. Application of this score to an elderly population has been shown to be an inconsistent predictor of mortality [12,16]. A study by Tornetta et al [17] addressed the factors affecting morbidity and mortality in elderly trauma patients. The study included 326 elderly trauma patients over the age of 60 who had suffered from blunt trauma. Mean age of the patients in the study was 72, mean ISS was 19.7 and overall mortality was 18%. Perdue et al found that mortality in elderly patients was twice that of younger patients with comparable ISS [18]. In our study population, we found that on univariate analysis ISS was significantly higher in the non-survivor group (28.6 ± 10.9 vs. 21.7 ± 7.1, p < 0.0001). However, on multivariate analysis ISS was not a predictor of in-hospital mortality.

Traumatic brain injury in geriatric patients has been recognized to result in a worse outcome when compared to younger counterparts, with a low admission GCS commonly recognized as a poor prognostic indicator [19]. Head injury was predominant in all our age groups with a homogenous head AIS distribution. It was strongly associated with in-hospital mortality (p < 0.0001) and associated with other predictors of mortality: low GCS upon arrival and the need for pre-hospital intubation (both p < 0.0001). This finding was not surprising and is strongly supported in the literature [19-21]. It appears that in the elderly population, age and perhaps neurologic status upon arrival, as reflected in the need for intubation and GCS, are more consistent predictors of in-hospital mortality.

Blood pressure and heart rate are not consistently the most sensitive indicators of physiologic distress in an elderly trauma patient. Given that a history of HTN and beta blockade use in the elderly population is not uncommon, these parameters may be unreliable. More so, these classic physiologic parameters are less reliable in the pre-hospital evaluation of the elderly patient, and consequently, an occult shock state may go unrecognized resulting in under-triage and evacuation to a non-trauma center [22]. Thus, there is a need for additional clinical assessment tools in the elderly population to aid clinicians in diagnosis.

In the elderly, pre-existing co-morbidities can adversely affect a patient's recovery from a traumatic injury [23]. This can be attributed to physiological changes related to age as well as pre-existing co-morbidities and poly-pharmacy [24,25]. Hollis et al have demonstrated that pre-existing medical conditions and increased age are independent risk factors of mortality after trauma [26]. We examined co-morbidities which are well-known to affect survival. On univariate analysis only an elevated creatinine level and a previous diagnosis of CRF were associated with in-hospital mortality. Multivariate analysis showed CRF as a predictor of in-hospital mortality (OR 3.65, p = 0.005). It appears that other common medical conditions such as HTN, ischemic heart disease, and diabetes mellitus, or the number of co-morbidities, do not affect in-hospital mortality.

In major trauma, mortality in the elderly was found to be nearly double compared to mortality in the younger age group [27] and the complication death rate to be markedly higher, especially for pulmonary and infectious complications. It has been previously reported in orthopedic trauma patients with typical femoral fractures that pulmonary and infectious complications are predominant [28,29]. In our population, pulmonary and infectious complications did not appear to affect survival. However, the total number of complications was higher in non-survivors compared with survivors (p = 0.04).

Elderly trauma patients are often admitted to the ICU despite their potentially stable appearance because of their high propensity to deteriorate rapidly. Early transfer from the ED to the ICU allows the performance of thorough and continuous hemodynamic monitoring. In this setting, aggressive and targeted management can prevent hypoperfusion, reduce the incidence of multiple organ failure, improve survival, and allow a rapid diagnostic workup. As expected, we found that the requirement for ICU was significantly higher in the mortality group (71.6% vs. 50.6%, p = 0.001). Additionally, ICU admission was significantly higher in the 60-69 age group (67.6%), mainly due to more severe MOI, often MVC. However, on multivariate analysis, ICU stay was not a predictor of in-hospital mortality.

Low-energy falls have been reported to account for only 9–11% of injury-related deaths in the general population. However, in patients over the age of 65 they account for more than 50% of traumatic deaths [30-32]. Our results showed that MOI and distribution of injury varied between the age groups. Greater than 80% of trauma admissions in the ≥80 group were caused by falls resulting primarily in head trauma (90%). This may be due to the poor overall condition of the frail, elderly patient who stumbles and loses balance at home. Although fall as MOI was significantly more common with increasing age, it did not affect in-hospital mortality.

### Limitations

Our analysis has several potential limitations. EMS crews in the Jerusalem district utilize age ≥ 60 as a criterion to transport patients to our level I trauma center, the only such center in the area. This study may have thus represented a population bias and therefore be limited in its ability to extrapolate our findings to other institutions. Patients were not analyzed based on hemodynamic status upon arrival, because criteria for hemodynamic instability are not clear and differ in age groups and individuals receiving medical treatment for pre-existing cardiovascular diseases. The presence of co-morbidities was based on data collection from the trauma registry with the intrinsic potential for error. Finally, this study was a retrospective review and is therefore susceptible to the inherent limitations.

## Conclusions

Current trauma scoring systems are insufficient in directing management and predicting survival for elderly injured patients. We found that advanced age, especially age ≥80, low GCS, elevated INR, pre-existing CRF and the requirement for intubation on admission are the strongest independent predictors of mortality in elderly population following severe trauma. Further research comprising age and other medical-related parameters will help formulate more predictive trauma scores and advanced treatment algorithms to facilitate treatment.

## Competing interests

The authors declare that they have no competing interests.

## Authors' contributions

MB: study conception, analysis and interpretation of data, drafting of manuscript. DW: study conception, analysis and interpretation of data, drafting of manuscript. DK: acquisition of data, analysis and interpretation of data. TBA: statistical analysis, analysis and interpretation of data. AIR: study conception, drafting of manuscript, critical revision. MAG: acquisition of data, analysis and interpretation of data. RE: statistical analysis, drafting of manuscript, critical revision. GA: study conception, analysis and interpretation of data, drafting of manuscript, critical revision. All authors read and approved the final manuscript.

## References

[B1] World health organization 10 facts on ageing and the life coursehttp://www.who.int/features/factfiles/ageing/en/index.html Assessed August 2013

[B2] State of Israel. Ministry of health Leading causes of death in Israel 2000-2010 http://www.health.gov.il/English/News_and_Events/Spokespersons_Messages/Pages/10032013_1.aspx. Ministry of health. Assessed Feb 2013

[B3] MacKenzie EJ Morris JA Smith GS Fahey M Acute hospital costs of trauma in the United States: implications for regionalized systems of care J Trauma 1990 21 1096 1103 10.1097/00005373-199009000-00005 2213943

[B4] Court-Brown CM Clement N Four score years and ten: an analysis of the epidemiology of fractures in the very elderly Injury 2009 21 1111 1114 10.1016/j.injury.2009.06.011 19596316

[B5] Rushing AM Scalea TM Trauma resuscitation of the elderly patient Clinics Geriatr 2010 21 34 36

[B6] Kuhne CA Ruchholtz S Kaiser GM Nast-Kolb D Working group on multiple trauma of the German society of trauma. Mortality in severely injured elderly trauma patients–when does age become a risk factor? World J Surg 2005 21 1476 1482 10.1007/s00268-005-7796-y 16228923

[B7] Probst C Zelle BA Sittaro NA Lohse R Krettek C Pape HC Late death after multiple severe trauma: when does it occur and what are the causes? J Trauma 2009 21 1212 1217 10.1097/TA.0b013e318197b97c 19359940

[B8] Davidson GH Hamlat CA Rivara FP Koepsell TD Jurkovich GJ Arbabi S Long-term survival of adult trauma patients JAMA 2011 21 1001 1007 10.1001/jama.2011.259 21386078

[B9] Grossman MD Ofurum U Stehly CD Stoltzfus J Long-term survival after major trauma in geriatric trauma patients: the glass is half full J Trauma Acute Care Surg 2012 21 1181 1185 22673243 10.1097/TA.0b013e31824d0e6d

[B10] Clement ND Tennant C Muwanga C Polytrauma in the elderly: predictors of the cause and time of death Scand J Trauma Resusc Emerg Med 2010 21 26 10.1186/1757-7241-18-26 20465806 PMC2880283

[B11] van Aalst JA Morris JA Jr Yates HK Miller RS Bass SM Severely injured geriatric patients return to independent living: a study of factors influencing function and independence J Trauma 1991 21 1096 1101 1875435

[B12] Oreskovich MR Howard JD Copass MK Carrico CJ Geriatric trauma: injury patterns and outcome J Trauma 1984 21 565 572 10.1097/00005373-198407000-00003 6748116

[B13] Jacobs DG Plaisier BR Barie PS Hammond JS Holevar MR Sinclair KE Scalea TM Wahl W EAST Practice Management Guidelines Work Group Practice management guidelines for geriatric trauma: the EAST practice management guidelines work group 2001 http://www.east.org/tpg/geriatric.pdf. Accessed 15 May 2013 10.1097/01.TA.0000042015.54022.BE 12579072

[B14] McMahon DJ Shapiro MB Kauder DR The injured elderly in the trauma intensive care unit Surg Clin North Am 2000 21 1005 1019 10.1016/S0039-6109(05)70110-9 10897275

[B15] Sikand M Williams K White C Moran CG The financial cost of treating polytrauma: implications for tertiary referral centres in the United Kingdom Injury 2005 21 733 737 10.1016/j.injury.2004.12.026 15910825

[B16] Sharma OP Oswanski MF Sharma V Stringfellow K Raj SS An appraisal of trauma in the elderly Am Surg 2007 21 354 358 17439028

[B17] Tornetta P 3rd Mostafavi H Riina J Turen C Reimer B Levine R Morbidity and mortality in elderly trauma patients J Trauma 1999 21 702 706 10.1097/00005373-199904000-00024 10217237

[B18] Perdue PW Watts DD Kaufmann CR Trask AL Differences in mortality between elderly and younger adult trauma patients: Geriatric status increases risk of delayed death J Trauma 1998 21 805 810 10.1097/00005373-199810000-00034 9783625

[B19] Utomo WK Gabbe BJ Simpson PM Cameron PA Predictors of in-hospital mortality and 6-month functional outcomes in older adults after moderate to severe traumatic brain injury Injury 2009 21 973 977 10.1016/j.injury.2009.05.034 19540490

[B20] Thompson HJ McCormick WC Kagan SH Traumatic brain injury in older adults: epidemiology, outcomes, and future implications J Am Geriatr Soc 2006 21 1590 1595 10.1111/j.1532-5415.2006.00894.x 17038079 PMC2367127

[B21] Timmons T Menaker J Traumatic brain injury in elderly Clinics Geriatr 2010 21 20 24

[B22] Salottolo KM Mains CW Offner PJ Bourg PW Bar-Or D A retrospective analysis of geriatric trauma patients: venous lactate is a better predictor of mortality than traditional vital signs Scand J Trauma Resusc Emerg Med 2013 21 7 10.1186/1757-7241-21-7 23410202 PMC3598961

[B23] Sasser SM Hunt RC Faul M Sugerman D Pearson WS Dulski T Wald MM Jurkovich GJ Newgard CD Lerner EB Centers for disease control and prevention (CDC). Guidelines for field triage of injured patients: recommendations of the national expert panel on field triage, 2011 MMWR Recomm Rep 2012 21 1 20 22237112

[B24] Niven DJ Kirkpatrick AW Ball CG Laupland KB Effect of comorbid illness on the long-term outcome of adults suffering major traumatic injury: a population-based cohort study Am J Surg 2012 21 151 156 10.1016/j.amjsurg.2012.02.014 22608670

[B25] Heffernan DS Thakkar RK Monaghan SF Ravindran R Adams CA Jr Kozloff MS Gregg SC Connolly MD Machan JT Cioffi WG Normal presenting vital signs are unreliable in geriatric blunt trauma victims J Trauma 2010 21 813 820 10.1097/TA.0b013e3181f41af8 20938267

[B26] Hollis S Lecky F Yates DW Woodford M The effect of pre-existing medical conditions and age on mortality after injury J Trauma 2006 21 1255 1260 10.1097/01.ta.0000243889.07090.da 17099538

[B27] Giannoudis PV Harwood PJ Court-Brown C Pape HC Severe and multiple trauma in older patients; incidence and mortality Injury 2009 21 362 367 10.1016/j.injury.2008.10.016 19217104

[B28] Liem IS Kammerlander C Raas C Gosch M Blauth M Is there a difference in timing and cause of death after fractures in the elderly? Clin Orthop Relat Res 2013 21 2846 2851 10.1007/s11999-013-2881-2 23460485 PMC3734403

[B29] Tan HB MacDonald DA Matthews SJ Giannoudis PV Incidence and causes of mortality following acute orthopaedic and trauma admissions Ann R Coll Surg Engl 2004 21 156 160 10.1308/003588404323043256 15140297 PMC1964185

[B30] Baker SP Harvey AH Fall injuries in the elderly Clin Geriatr Med 1985 21 501 512 3913506

[B31] Hogue CC Injury in late life. Part I. Epidemiology J Am Geriatr Soc 1982 21 183 190 7037915 10.1111/j.1532-5415.1982.tb01302.x

[B32] Shortt NL Robinson CM Mortality after low-energy fractures in patients aged at least 45 years old J Orthop Trauma 2005 21 396 400 10.1097/01.bot.0000155311.04886.7e 16003199

